# Hippocampal Damage Increases Deontological Responses during Moral Decision Making

**DOI:** 10.1523/JNEUROSCI.0707-16.2016

**Published:** 2016-11-30

**Authors:** Cornelia McCormick, Clive R. Rosenthal, Thomas D. Miller, Eleanor A. Maguire

**Affiliations:** ^1^Wellcome Trust Centre for Neuroimaging, Institute of Neurology, University College London, London WC1N 3BG, United Kingdom, and; ^2^Nuffield Department of Clinical Neurosciences, University of Oxford, Oxford OX3 9DU, United Kingdom

**Keywords:** amnesia, hippocampus, moral judgements, scene construction, utilitarian, vmPFC

## Abstract

Complex moral decision making is associated with the ventromedial prefrontal cortex (vmPFC) in humans, and damage to this region significantly increases the frequency of utilitarian judgments. Since the vmPFC has strong anatomical and functional links with the hippocampus, here we asked how patients with selective bilateral hippocampal damage would derive moral decisions on a classic moral dilemmas paradigm. We found that the patients approved of the utilitarian options significantly less often than control participants, favoring instead deontological responses—rejecting actions that harm even one person. Thus, patients with hippocampal damage have a strikingly opposite approach to moral decision making than vmPFC-lesioned patients. Skin-conductance data collected during the task showed increased emotional arousal in the hippocampal-damaged patients and they stated that their moral decisions were based on emotional instinct. By contrast, control participants made moral decisions based on the integration of an adverse emotional response to harming others, visualization of the consequences of one's action, and the rational re-evaluation of future benefits. This integration may be disturbed in patients with either hippocampal or vmPFC damage. Hippocampal lesions decreased the ability to visualize a scenario and its future consequences, which seemed to render the adverse emotional response overwhelmingly dominant. In patients with vmPFC damage, visualization might also be reduced alongside an inability to detect the adverse emotional response, leaving only the utilitarian option open. Overall, these results provide insights into the processes involved in moral decision making and highlight the complementary roles played by two closely connected brain regions.

**SIGNIFICANCE STATEMENT** The ventromedial prefrontal cortex (vmPFC) is closely associated with the ability to make complex moral judgements. When this area is damaged, patients become more utilitarian (the ends justify the means) and have decreased emotional arousal during moral decision making. The vmPFC is closely connected with another brain region—the hippocampus. In this study we found that patients with selective bilateral hippocampal damage show a strikingly opposite response pattern to those with vmPFC damage when making moral judgements. They rejected harmful actions of any kind (thus their responses were deontological) and showed increased emotional arousal. These results provide new insights into the processes involved in moral decision making and highlight the complementary roles played by two closely connected brain regions.

## Introduction

The hippocampus and ventromedial prefrontal cortex (vmPFC) are often coactivated during neuroimaging studies that involve such processes as autobiographical memory recall (Svoboda et al., 2006), theory of mind (Spreng et al., 2009), and constructing scenes and events in the imagination (Hassabis and Maguire, 2009). However, the precise contributions of the hippocampus and vmPFC to these tasks remain unclear. One way to try to dissociate their functional roles is to administer tasks typically associated with one of these brain areas to patients with selective damage to the other brain area. For example, the ability to mentally construct scenes, a process typically associated with the hippocampus (Hassabis et al., 2007), has recently been shown to be impaired in patients with vmPFC damage (Bertossi et al., 2016). A classic hallmark of vmPFC damage is altered moral decision making (Ciaramelli et al., 2007, 2012; Koenigs et al., 2007; Thomas et al., 2011). Therefore, the goal of the current study was to examine moral decision making in patients with selective bilateral hippocampal damage.

The moral dilemmas task is an established means by which to examine moral decision making. Participants read a short scenario and make moral decisions about what action they personally would take (Greene et al., 2001). Critically, the vmPFC seems necessary for only one type of dilemma, namely those scenarios typically described as being very personal and emotionally charged (Ciaramelli et al., 2007; Koenigs et al., 2007). Even for healthy controls, dealing with these personal dilemmas is difficult, as reflected in increased reaction times (Ciaramelli et al., 2007; Terbeck et al., 2013) and in skin conductance responses (Moretto et al., 2010). Koenigs et al. (2007) further dissociated the personal dilemmas into two subgroups, low-conflict and high-conflict dilemmas. Only in the high-conflict dilemmas is one required to decide between killing one person to save the lives of others (i.e., a utilitarian response) or never killing anyone (i.e., a deontological response) but being responsible for the deaths of multiple people (Conway and Gawronski, 2013). These high-conflict dilemmas create an internal tension between the emotional rejection of harming somebody and the logical desire to save lives (Shenhav and Greene, 2014; Wojciszke et al., 2015). According to the dual-process theory of moral judgments (Greene et al., 2004; Greene, 2007), the vmPFC is crucial for detecting the intuitive emotional rejection of harming someone. Therefore, following vmPFC damage, one is left with only the rational, utilitarian response as an option (Ciaramelli et al., 2007; Greene, 2007; Koenigs et al., 2007; Bertossi et al., 2016).

It is unclear how patients with selective bilateral hippocampal damage would approach these high-conflict dilemmas. They are able to think rationally, demonstrating intact high-level causal reasoning, counterfactual thinking (Mullally and Maguire, 2014), basic emotional processing (Clark and Maguire, 2016), and theory of mind (Rosenbaum et al., 2007). Consequently, hippocampal-damaged patients might not differ from controls. Conversely, patients with hippocampal damage show reduced empathy (Beadle et al., 2013), possibly due to a diminished ability to vividly construct another person's situation (Clark and Maguire, 2016). In fact, visualization of scenarios alters moral decision making in healthy controls (Amit and Greene, 2012), suggesting a possibly important role for scene construction in this task. Thus, patients with hippocampal damage might respond differently to moral scenarios compared with control participants.

To fully explore moral decision making during the dilemmas task, we also acquired skin conductance as a measure of emotional arousal (Tranel and Damasio, 1994; Bechara et al., 1997) and added following the task a debriefing that probed the strategies participants used to make their decisions. Finally, to assess possible personality changes in general following hippocampal damage, close relatives of the patients completed the Iowa Scales of Personality Change (Barrash et al., 2000, 2011). This questionnaire is commonly used to assess changes in emotional functioning and social behaviors in patients with vmPFC damage.

## Materials and Methods

### 

#### Participants

Five patients (all males; mean age, 54.4 years; age range, 27–70 years) with selective bilateral hippocampal lesions due to voltage-gated potassium channel (VGKC)-complex antibody-mediated limbic encephalitis (LE) were tested (Table 1, demographics). Patients with VGKC-complex LE present with amnesia and seizures, followed by a non-reversible atrophy typically confined to the hippocampus (Dalmau and Rosenfeld, 2014; see also Maguire et al., 2016 for further discussion of hippocampal damage in this condition). In line with previous studies that have studied the chronic VGKC-complex LE phenotype (Frisch et al., 2013), the patients included here displayed selective memory impairment on tests of immediate and delayed recall, and they had significantly fewer internal (episodic) details but not external (semantic) details compared with controls on the Autobiographical Interview (Levine et al., 2002; Table 2). Deficits were not apparent in any other cognitive or emotional domain, including mood, executive functions, language, and perceptual abilities (Table 2).

**Table 1.** Summary of demographic profile

Patient ID	Gender	Handedness	Age (years)	Wechsler Abbreviated Scale of Intelligence (Wechsler, 1999)		Chronicity	Hippocampus	
				Scaled scores from Matrix Reasoning subtest	Scaled scores from Similarities subtest		Left volume	Right volume
HC01	Male	Right	68	16	15	8	2759	3308
HC02	Male	Right	70	12	13	9	2607	2755
HC04	Male	Right	27	14	11	9	2819	2804
HC07	Male	Right	48	15	15	5	2610	2702
HC08	Male	Right	59	12	12	4	2506	2803
HC group	5 (males)	5 (right)	Mean, 54.4; SD, 17.6	Mean, 13.8; SD, 1.8	Mean, 13.2; SD, 1.8	Mean, 7.0; SD, 2.3	Mean, 2660; SD, 126	Mean, 2874; SD, 245
CTL group	11 (males)	10 (right)	Mean, 57.1; SD, 17.3	Mean, 14.0; SD, 1.5	Mean, 12.0; SD, 2.6	Not applicable	Mean, 3191; SD, 357	Mean, 3277; SD, 335
*p* value of two-sample *t* test			0.78	0.82	0.37		0.008*^a^*	0.037*^a^*

*^a^* Significant difference.

**Table 2.** Summary of neuropsychological profile

Group	Autobiographical Interview scores*^b^*		Immediate recall memory*^c^*	Delayed recall memory*^d^*	Recognition memory*^e^*	Semantic memory*^f^*	Working memory*^g^*	Language abilities*^h^*	Executive functions*^i^*	Perception*^j^*	Mood*^k^*
	Internal (episodic) details	External (semantic) details									
Hippocampal-damaged patients											
Mean	33.0	5.7	−0.4	−0.6	−0.1	0.3	−0.3	0.0	−0.3	−0.4	0.1
SD	6.5	4.1	0.4	0.8	1.2	0.8	0.9	0.8	0.5	1.7	1.1
Healthy control participants											
Mean	51.3	5.9	0.3	0.3	0.1	0.1	0.2	0.2	0.1	0.2	0.1
SD	13.6	2.2	0.3	0.6	0.6	0.8	1.1	0.9	0.6	0.3	0.8
*p* value of two-sample *t* test	0.02*^a^*	0.94	0.003*^a^*	0.04*^a^*	0.59	0.66	0.37	0.66	0.19	0.3	0.95

With the exception of the Autobiographical Interview scores, which are shown as standard means, scores (where available scaled scores) of individual tests have been transformed into *z*-scores and averaged across patients and controls within each neuropsychological domain. Therefore, a mean *z*-score of zero indicates that both groups had the same mean.

*^a^* Significant difference.

*^b^* Autobiographical memory performance of the patients (Levine et al., 2002) was compared to a separate control group (5 males; 1 female; mean age, 55.2 ± 18 years; range, 22–69 years; all right-handed).

*^c^* Wechsler Memory Scale (WMS-III; Wechsler, 1997), logical memory 1 units and thematic scores, word list 1 total recall, and Rey-Osterrieth complex figure (Osterrieth, 1944) immediate recall.

*^d^* WMS-III logical memory 2 units and thematic scores, and Rey-Osterrieth complex figure delayed recall.

*^e^* Warrington Recognition Memory Test for words and faces (Warrington, 1984), WMS-III word list 2 recognition.

*^f^* Warrington Graded Naming Test (McKenna and Warrington, 1980; Warrington, 2010).

*^g^* WMS-III digit span subtest.

*^h^* Delis-Kaplan Executive Function System (D-KEFS; Delis et al., 2001) letter fluency and category fluency tests.

*^i^* D-KEFS category switch test, word-color interference test, trails test (average of visual scanning, number sequencing, letter sequencing, number–letter switching, and motor speed tests), Hayling Test (Burgess and Shallice, 1997) Sentence Completion Test.

*^j^* Visual Object and Space Perception Battery (Warrington and James, 1991) dot counting, cube analysis, position discrimination tests, and the Rey-Osterrieth Complex Figure copy.

*^k^* Hospital Anxiety and Depression Scale (Zigmond and Snaith, 1983).

Eleven control participants (all males; mean age, 57.1 years; age range, 25–77 years) who were closely matched to the patients on sex, age, and general intellectual ability (Table 1), were also tested.

Each participant gave informed written consent to participation in accordance with the approval of the local research ethics committee. For patients, close relatives were also involved in the consenting process.

#### Characterization of hippocampal damage

All participants underwent structural MR imaging using a 3.0 T whole-body MR scanner (Magnetom TIM Trio, Siemens Healthcare) operated with a radio frequency (RF) transmit body coil and 32-channel head RF receive coil.

In the first instance, imaging was limited to a partial volume focused on the temporal lobes. These structural images were collected using a single-slab 3D T2-weighted turbo spin echo sequence with variable flip angles (SPACE; Mugler et al., 2000) in combination with parallel imaging, to simultaneously achieve a high image resolution of ∼500 μm, high sampling efficiency, and short scan time while maintaining a sufficient signal-to-noise ratio (SNR). After excitation of a single axial slab, the image was read out with the following parameters: resolution, 0.52 × 0.52 × 0.5 mm^3^; matrix, 384 × 328; partitions, 104; partition thickness, 0.5 mm; partition oversampling, 15.4%; field of view, 200 × 171 mm^2^; TE = 353 ms; TR = 3200 ms; generalized autocalibrating partially parallel acquisitions (GRAPPA) × 2 in phase-encoding (PE) direction; bandwidth, 434 Hz/pixel; echo spacing, 4.98 ms; turbo factor in PE direction, 177; echo train duration, 881. *K*-space averaging was used to boost SNR with 90% resampling (i.e., average factor, 1.9) weighted to the center of *k*-space. For reduction of signal bias due to, for example, spatial variation in coil sensitivity profiles, the images were normalized using a prescan, and a weak intensity filter was applied as implemented by the scanner's manufacturer. It took 12 min to obtain a scan. To improve the SNR of the anatomical image, two or three scans were acquired for each participant.

**Figure 1.**
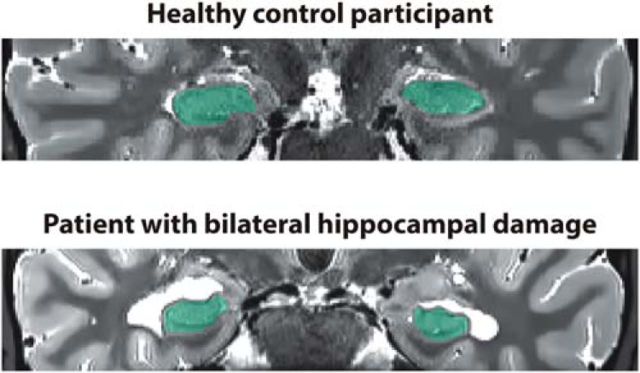
Characterization of hippocampal damage. Example T2-weighted coronal structural MR images of a healthy control participant (top) and a patient with bilateral hippocampal damage (bottom). The hippocampi are marked in green. Images are displayed in native space corresponding approximately to the position of *y* = −10 in the Montreal Neurological Institute coordinate system.

Images from each participant were coregistered and denoised following the Rician noise estimation (Coupé et al., 2010). The denoised images were averaged and smoothed with a full-width at half-maximum kernel of 2 × 2 × 2 mm. Left and right hippocampi were then manually (and blindly) segmented and volumes extracted using the ITK Snap software version 3.4.0 (Yushkevich et al., 2006). As detailed on Table 1 (Fig. 1), we found pronounced hippocampal volume loss in patients compared with the control participants on the left [patients with hippocampal damage (HC), 2660 ± 126 mm^3^ (mean ± SD); control participants (CTL), 3191 ± 357 mm^3^; *t*_(12)_ = 3.2, *p* = 0.008, Cohen's *d* = 2.0] and right (HC, 2874 ± 245 mm^3^; CTL, 3277 ± 335 mm^3^; *t*_(12)_ = 2.3, *p* = 0.037, Cohen's *d* = 1.4).

To exclude group differences in moral decision making based on potential differences in amygdalae volumes, left and right amygdalae were also manually (and blindly) segmented following standard protocols (Pruessner et al., 2000; Entis et al., 2012). Volumes of left (HC, 1403 ± 202 mm^3^; CTL, 1310 ± 217 mm^3^; *t*_(12)_ = 0.79, *p* = 0.45) and right (HC, 1422 ± 319 mm^3^; CTL, 1308 ± 246 mm^3^; *t*_(12)_ = 0.75, *p* = 0.47) amygdalae did not differ between patients and controls.

To rule out consistent differences between patients and controls in gray matter volume elsewhere in the brain (e.g., in the vmPFC) we also collected a whole-brain structural T1-weighted sequence at an isotropic resolution of 800 μm (Callaghan et al., 2015) using a field of view of 256 mm head–foot, 224 mm anterior–posterior (AP), and 166 mm right–left (RL) on which to conduct a voxel-based morphometry (VBM) analysis. This was a spoiled multiecho 3D fast low-angle shot acquisition with a flip angle of 21° and a repetition time (TR) of 25 ms. To accelerate the data acquisition, partially parallel imaging using the GRAPPA algorithm was used in each PE direction (AP and RL) with 40 reference lines and a speed-up factor of two. Gradient echoes were acquired with alternating readout polarity at eight equidistant echo times ranging from 2.34 to 18.44 ms in steps of 2.30 ms using a readout bandwidth of 488 Hz/pixel (Helms and Dechent, 2009). The first six echoes were averaged to increase SNR (Helms and Dechent, 2009), producing a T1-weighted image with an effective echo time of 8.3 ms, which was used for the VBM analysis.

An automated VBM analysis was performed using SPM12 (Statistical Parametric Mapping, Wellcome Trust Centre for Neuroimaging, London, UK). The averaged T1-weighted images were segmented into gray and white matter probability maps using the unified segmentation approach (Ashburner and Friston, 2005). Intersubject registration of the tissue classes was performed using Dartel, a non-linear diffeomorphic algorithm (Ashburner, 2007). The resulting Dartel template and deformations were used to normalize the tissue probability maps to the stereotactic space defined by the Montreal Neurological Institute template. For VBM analysis, the normalization procedure included modulating the gray matter tissue probability maps by the Jacobian determinants of the deformation field and smoothing with an isotropic Gaussian smoothing kernel of 8 mm full width at half maximum. The normalized gray matter from controls and the patients with hippocampal damage were contrasted using a two-sample *t* test and thresholded at *p* < 0.001 uncorrected and a cluster extend of 50 voxels. This whole-brain analysis revealed no significant differences outside of the hippocampus between the gray matter volumes of healthy controls and patients.

#### Moral dilemmas task

The main focus of this study was to assess how patients with bilateral hippocampal damage would perform on a moral dilemmas task usually associated with an increased number of utilitarian responses in patients with vmPFC damage. We therefore opted to administer the moral dilemmas task following as closely as possible the procedure described by Koenigs et al. (2007).

The task consisted of 50 hypothetical scenarios originally based on a study by Greene et al. (2001) but adapted by Koenigs et al. (2007). For the scenarios, we used the exact wording from the supplementary material (Koenigs et al., 2007), just changing American to British references (e.g., dollars to pounds, etc.).

Each scenario was presented on three consecutive text slides on a computer screen. The first two text slides described the scenario and the third slide always posed a question about an action related to the scenario in the form of “Would you…, to…?”. Participants pressed an “up” arrow key to advance to the next slide, and a “yes” and “no” button for the third slide. For the first two slides, there was no time limit and for the third slide there was a time limit of 25 s to read the question and respond. The scenarios were presented in a pseudorandom order so that never more than two scenarios of the same class were presented consecutively. Further, the task was divided into five sessions each containing 10 scenarios. The breaks between sessions (no more than 3 min) were just long enough to ensure that the participants were able to continue with the task and remembered the instructions.

The scenarios fell into the four classes based on previous studies (Greene et al., 2001; Koenigs et al., 2007). These were (1) non-moral scenarios, (2) impersonal scenarios, (3) personal low-conflict scenarios, and (4) personal high-conflict scenarios.

##### Non-moral scenarios.

In the non-moral scenarios (*n* = 18), participants had to follow the logic of a story without a moral dimension. For example: “You are a farm worker driving a turnip-harvesting machine. You are approaching two diverging paths (slide 1). By choosing the path on the left you will harvest ten bushels of turnips. By choosing the path on the right you will harvest twenty bushels of turnips. If you do nothing your turnip-harvesting machine will turn to the left (slide 2). Would you turn your turnip-harvesting machine to the right to harvest twenty bushels of turnips instead of ten (slide 3)?”

##### Impersonal scenarios.

In the impersonal scenarios (*n* = 11), participants had to make moral judgments that are more intuitively impersonal and less emotional. A common example for this category is often the standard trolley scenario in which one has to turn a switch to divert a runaway trolley from the tracks with five people on it toward a track with only one person on it. Many of these impersonal dilemmas, however, do not involve the death of people and describe actions that are milder. For example: “You are walking down the street when you come across a wallet lying on the ground. You open the wallet and find that it contains several hundred pounds in cash as well as the owner's driver's license (slide 1). From the credit cards and other items in the wallet it's very clear that the wallet's owner is wealthy. You, on the other hand, have been hit by hard times recently and could really use some extra money. You consider sending the wallet back to the owner without the cash, keeping the cash for yourself (slide 2). Would you keep the money you found in the wallet to have more money for yourself (slide 3)?”

##### Personal low-conflict scenarios.

In the personal low-conflict scenarios (*n* = 8), participants had to make moral judgments that are personal and highly emotional. According to Koenigs et al. (2007), these are scenarios that typically result in rejection of the outlined action by healthy participants and patients with vmPFC damage. Most of these scenarios do not involve the loss of lives but contain inexcusable, self-beneficial outcomes as a consequence of quite terrible actions. For example: “You are driving along a country road when you hear a plea for help coming from some roadside bushes. You pull over and encounter a man whose legs are covered with blood. The man explains that he has had an accident while hiking and asks you to take him to a nearby hospital (slide 1). Your initial inclination is to help this man, who will probably lose his leg if he does not get to the hospital soon. However, if you give this man a lift, his blood will ruin the leather upholstery of your car (slide 2). Would you leave this man by the side of the road to preserve your leather upholstery (slide 3)?”

##### Personal high-conflict scenarios.

In the personal high-conflict scenarios (*n* = 13), participants had to make moral judgments that are personal, highly emotional, and controversial. These scenarios typically do not receive unanimous rejection from healthy participants. Importantly, for this category of moral dilemma, patients with vmPFC damage, compared with controls, approved of the utilitarian option significantly more often (Koenigs et al., 2007). For most of these scenarios, the moral question is whether to take a life with your own hands to save the lives of others. For example: “You are the captain of a military submarine, traveling underneath a large iceberg. An onboard explosion has caused you to lose most of your oxygen supply and has injured one of your crew who is quickly losing blood. The injured crew member is going to die from his wounds no matter what happens (slide 1). The remaining oxygen is not sufficient for the entire crew to make it to the surface. The only way to save the other crew members is to shoot dead the injured crew member so that there will be just enough oxygen for the rest of the crew to survive (slide 2). Would you kill the fatally injured crew member to save the lives of the remaining crew members (slide 3)?”

#### Galvanic skin responses

We used skin conductance as a dependent measure of emotional arousal and autonomic activity in response to the scenarios (Tranel and Damasio, 1994; Bechara et al., 1997). For each participant, skin conductance was recorded in a magnetically shielded room on the volar surface of the middle and index finger of the left hand using disposable Ag/AgCl-laminated electrodes (EL509 RT dry electrodes, Biopac Systems) and isotonic recording electrode gel (Gel 101, Biopac Systems). This allowed the dominant right hand to be free for performing the button presses necessary for the task. The electrodes were attached to a custom-built constant-voltage coupler (2.5 V), based on a differential amplifier and DC-powered by a 12 V battery. The output of the coupler was converted into an optical pulse frequency. This pulse signal was transmitted using an optical fiber, digitally converted outside the shielded room with 2 μs time resolution (Micro1401, Cambridge Electronic Design), and recorded (Spike2, Cambridge Electronic Design). Stimulus onset was signaled by transistor–transistor logic pulses via the stimulus computer's parallel port, and corresponded to the onset of the three text slides and the final response to the question on slide three. Thus, for each scenario, four time points were marked with triggers.

Data analysis was performed using SCRalyze (Bach et al., 2009) following the general linear convolution model for evoked skin conductance responses. This protocol included bandpass filtering (first-order Butterworth filter, 0.0159 and 5 Hz cutoff frequencies, sequentially forward and backward), downsampling the data to 10 Hz, and normalizing the data to avoid between-subject differences in response amplitude due to peripheral factors, such as skin properties. Data were then concatenated over the five sessions, and parameter estimates were calculated for each condition. Since each scenario was associated with four marked time points (i.e., slides 1, 2, 3, and the final response) and scenarios were sorted into four categories (i.e., non-moral, impersonal, personal low-conflict, and personal high-conflict), there were 16 parameter estimates for each participant. The resulting β values were then averaged across time points for each dilemma category and exported for statistical analyses.

#### Debriefing

As evident from the examples, some of these scenarios posed very difficult moral questions. It was important for us to capture the emotional impact of performing the task and the cognitive strategies used to make the moral decisions. Therefore, we asked each participant the following debriefing questions immediately after the conclusion of the task: (1) Did you have any problems keeping track of the story line of the scenarios? (Here, we aimed to assess whether the patients experienced difficulties related to their memory problem); (2) What was your strategy in responding to the scenarios? In particular, how did you decide for the sacrificial scenarios? Did this differ in any way from the other scenarios? How emotional did you find these scenarios?; (3) Did you imagine a scene in your mind's eye? Did that differ between non-sacrificial and sacrificial ones?; (4) Did you know any of the scenarios from before the experiment? (All participants answered “no” to this question).

#### Iowa Scales of Personality Change

To gain insight into whether personality changes usually associated with vmPFC lesions are also present in patients with hippocampal damage, we asked close relatives of the patients to complete the Iowa Scales of Personality Change (ISPC). This questionnaire consists of 30 scales. Twenty-six of these scales assess personality characteristics shown to change after vmPFC damage, including emotional functioning, behavioral control, social and interpersonal behavior, and higher-order cognitive abilities, such as decision making and insight (Barrash et al., 2000, 2011). In addition, there are four scales that are not associated with acquired brain damage that act as control scales to detect possible response bias. These scales measured frugality, vanity, manipulativeness, and type-A behavior. In the questionnaire, each characteristic is introduced by a brief description, followed by two rating scales. The relative is asked to rate the behavior before and after (so how it is now) the onset of hippocampal damage. Both ratings are made along a seven-point rating scale, with three being the average or usual level of a typical adult, and with higher ratings reflecting an increasing level of that behavior. Points of the scale are accompanied by example statements that help the relative choose an appropriate level. Importantly, relatives are instructed to rate exclusively the patient's behavior regardless of their belief about why the characteristic is at its current level.

The sensitive measure of the ISPC is based on the change scores calculated as the difference between premorbid and postmorbid level ratings. In our analysis, we excluded two scales that only assessed the postmorbid stage and therefore did not yield change scores. For all other 24 scales, we calculated the difference between postmorbid and premorbid level ratings. Positive values (maximum, 6) therefore reflect an increase and negative values (maximum, −6) reflect a decrease in a particular behavior.

We were also interested in whether personality traits differed between patients with vmPFC and hippocampal damage. To illustrate the main differences, an exploratory analysis was conducted to compare the change values of our patient cohort with the previously published change scores for patients with vmPFC damage (Barrash et al., 2000). Of note, in this latter study, relatives had to indicate a direct change measure on a five-point scale, whereas the current study used before and after ratings on a seven-point scale to calculate the change score.

#### Data analyses

Statistical significance levels of response proportion, reaction times, and skin conductance were assessed using separate two-way repeated-measures ANOVA (2way-RM-ANOVA) with participant group (patients, controls) as a factor with two levels and classes of moral dilemma as a repeated-measurement factor with four levels (non-moral, impersonal, personal-low, and personal-high). Main effects and interaction effects were evaluated first and a two-sided *p* value of <0.05 was used as a threshold to reject the null hypotheses in each case. Where 2way-RM-ANOVAs yielded significant main or interaction effects, we conducted *post hoc* comparisons between groups and dilemma classes using Sidak's multiple-comparison tests, again considering *p* values <0.05 as statistically significant.

Independent pairwise comparisons between the two groups (e.g., hippocampal volumes, neuropsychological test scores) were assessed using the Student's two-sample *t* test. Again, a two-sided *p* value of <0.05 was used as the threshold to reject the null hypotheses in each case.

We also report the effect sizes (using Cohen's *d*) and, where appropriate, show the data of every participant.

We explored the personality change scores from our patients with hippocampal damage and those of patients with vmPFC damage assessed with the ISPC. For patients with vmPFC damage, we only had access to sample size, mean, and SD (Barrash et al., 2000). Here, we conducted a 2way-ANOVA (2 groups × 28 personality traits). First, the significance of main effects and interaction term was assessed as before using a two-sided *p* value of <0.05. Since the interaction term reached significance, we performed pairwise comparisons using Fisher's least significant difference test between the means of both groups for each personality trait, again considering a two-sided *p* value of <0.05 a significant result. In this exploratory analysis, we opted not to correct for multiple comparisons since each mean was only compared with that of the other group for a specific personality trait and therefore never entered a comparison more than once.

## Results

### Moral dilemmas task

**Figure 2.**
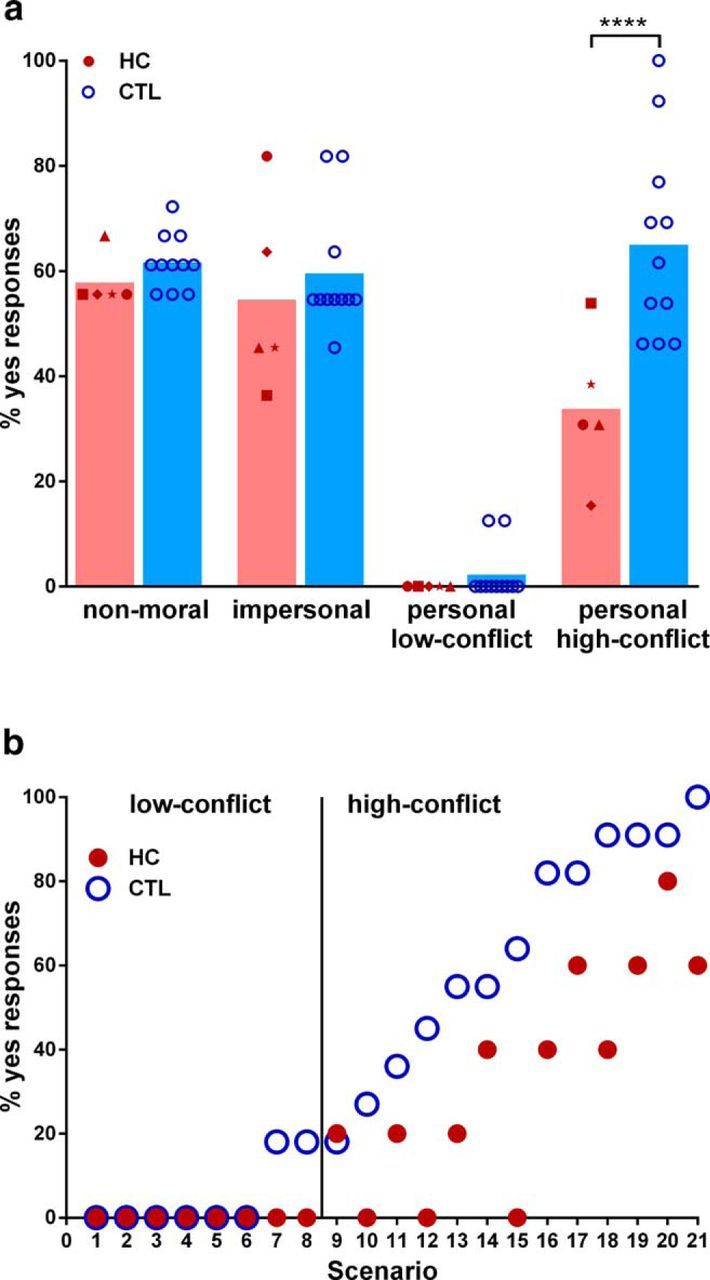
Moral judgments for each scenario class. ***a***, The percentage of “yes” responses for individual patients with hippocampal damage (HC, red symbols) and healthy control participants (CTL, blue circles). The height of the bars represents the mean. On personal high-conflict scenarios, patients with hippocampal damage, compared to healthy controls, responded significantly less often with “yes” (****p < 0.0001). ***b***, Percentage of “yes” responses are shown for each personal scenario given by patients with hippocampal damage (red symbols) and by healthy controls (blue circles). Individual scenarios are ordered by increasing frequency of “yes” responses by healthy controls. Responses did not differ for the low-conflict scenarios. For the majority of high-conflict scenarios, patients with hippocampal damage responded less often with “yes” compared with the healthy controls.

Figure 2*a* illustrates the proportion of yes responses to each of the four scenario classes for patients with bilateral hippocampal damage and matched healthy control participants. We found a significant main effect of group (*F*_(1,14)_ = 7.98, *p* = 0.013) and scenario class (*F*_(3,42)_ = 88.99, *p* = 0.0001), and an interaction effect between group and scenario class (*F*_(3,42)_ = 5.61, *p* = 0.0025).

There were no differences between the groups in the proportion of yes responses for non-moral (HC, 57.8 ± 4.9; CTL, 61.6 ± 5.2; *t*_(56)_ = 0.61, *p* = 0.96), impersonal (HC, 54.5 ± 18.2; CTL, 59.5 ± 11.8; *t*_(56)_ = 0.79, *p* = 0.9), and personal low-conflict scenarios (HC, 0.0 ± 0.0; CTL, 2.7 ± 5.1; *t*_(56)_ = 0.36, *p* = 0.99), indicating that the patients did not have difficulties following the logic of the scenarios and they were able to keep the information in their short-term memory to respond adequately and appropriately.

Interestingly, for the personal high-conflict scenarios, the patients approved the utilitarian option (i.e., proportion of yes responses) significantly less often than did the healthy controls (HC, 33.8 ± 13.9; CTL, 65.0 ± 18.6; *t*_(56)_ = 4.97, *p* = 0.0001, Cohen's *d* = 1.90). Whereas each low-conflict scenario resulted in strong rejections from patients and controls alike, the patients were specifically much less likely than control participants to approve the utilitarian option for the vast majority of the high-conflict scenarios (Fig. 2*b*).

It is interesting to contrast the moral decision making of our hippocampal-damaged patients to that of the previously reported behavior of patients with vmPFC damage [from Koenigs et al., 2007; vmPFC patients: *n* = 6; 3 males; mean age, 59.2 years old (SD 8.7); all right-handed; mean years of education, 12.5 (SD, 1.9)]. From this perspective, the low utilitarian response rate of our patients is especially interesting since it was these high-conflict scenarios that patients with vmPFC damage approved more often than healthy controls. In fact, contrasting the approval rate of the current hippocampal-damaged patients directly to that of patients with vmPFC lesions demonstrates the clear difference between the two patient cohorts. Patients with vmPFC damage approved the utilitarian option more often than patients with hippocampal damage (HC, 33.8 ± 13.9; vmPFC, 70.5 ± 29.4; *t*_(9)_ = 2.6, *p* = 0.03, Cohen's *d* = 1.61).

### Reaction times

There was a significant main effect of scenario class (*F*_(3,42)_ = 26.9, *p* = 0.0001) but no main effect of group (*F*_(1,14)_ = 3.8, *p* = 0.072) or interaction effect (*F*_(3,42)_ = 0.23, *p* = 0.8). Hence, both patients and controls showed the same pattern of reaction times across the four scenario classes. As expected, both groups spent longer on the personal high-conflict than on the non-moral (baseline) scenarios (HC: non-moral, 27.8 ± 8.9 s; personal high, 36.4 ± 10.1 s; *t*_(42)_ = 4.74, *p* = 0.001, Cohen's *d* = 0.9; CTL: non-moral, 22.3 ± 4.3 s; personal high, 30.1 ± 7.9 s; *t*_(42)_ = 6.32, *p* < 0.0001, Cohen's *d* = 1.3), impersonal (HC: impersonal, 29.8 ± 8.3 s; personal high, 36.4 ± 10.1 s; *t*_(42)_ = 3.6, *p* = 0.005, Cohen's *d* = 0.7; CTL: impersonal, 22.9 ± 5.6 s; personal high, 30.1 ± 7.9 s; *t*_(42)_ = 5.9, *p* < 0.001, Cohen's *d* = 1.1), and personal low-conflict scenarios (HC: personal low, 28.1 ± 5.9 s; personal high, 36.4 ± 10.1 s; *t*_(42)_ = 4.5, *p* < 0.001, Cohen's *d* = 1.0; CTL: personal low, 21.1 ± 4.2 s; personal high, 30.1 ± 7.9 s; *t*_(42)_ = 7.3, *p* < 0.001, Cohen's *d* = 1.4).

### Galvanic skin responses

**Figure 3.**
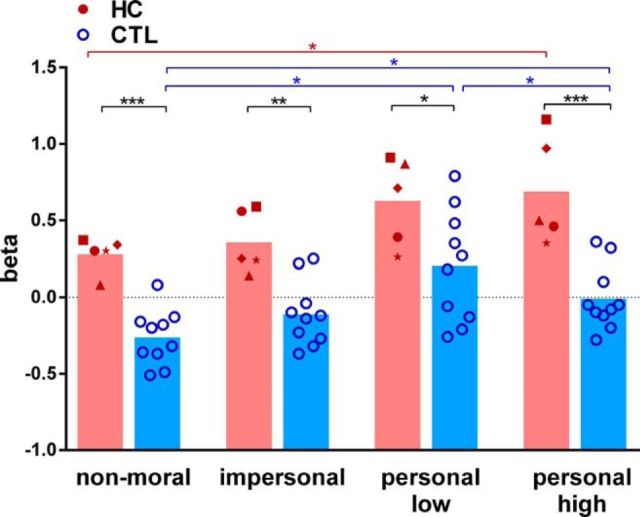
Skin conductance responses for each scenario class. Skin conductance responses are shown for individual patients with hippocampal damage (HC, red symbols) and healthy control participants (CTL, blue circles). The height of the bars represents the mean. *p < 0.05; **p < 0.01; ***p < 0.001. Between-group effects are indicated in black; within-group effects are indicated in color (HC in red, CTL in blue). Both groups reacted with increased skin conductance in response to the personal high-conflict compared with the non-moral (baseline) scenarios. In addition, hippocampal-damaged patients showed increased skin conductance to all scenario classes relative to the healthy controls.

In addition to behavioral responses, we also acquired skin conductance responses during task performance. Figure 3 illustrates the averaged β values for the four scenario classes for patients and controls. We were unable to collect skin conductance responses from one control participant because of technical problems. The skin conductance results below are therefore based on five patients with hippocampal damage and 10 healthy control participants.

We found a main effect of scenario class (*F*_(3,39)_ = 9.8, *p* = 0.0001) and group membership (*F*_(1,13)_ = 36.2, *p* = 0.0001) but no interaction effect (*F*_(3,39)_ = 0.58, *p* = 0.63). Both patients and controls showed increased skin conductance responses for the personal high-conflict compared with the non-moral (baseline) scenarios (HC: non-moral, 0.28 ± 0.1; personal high, 0.68 ± 0.3; *t*_(39)_ = 2.9, *p* = 0.041, Cohen's *d* = 1.8; CTL: non-moral, −0.26 ± 0.2; personal high, −0.01 ± 0.2; *t*_(39)_ = 3.1, *p* = 0.025, Cohen's *d* = 0.8). In addition, controls also showed an increased response for the personal low-conflict compared with the non-moral (CTL: non-moral, −0.26 ± 0.2; personal low, 0.21 ± 0.4; *t*_(39)_ = 4.6, *p* = 0.003, Cohen's *d* = 1.5) and impersonal scenarios (CTL: impersonal, −0.11 ± 0.2; personal low, 0.21 ± 0.4; *t*_(39)_ = 3.1, *p* = 0.022, Cohen's *d* = 1.0).

Interestingly, compared with the healthy control participants, the patients showed increased skin conductance responses for all scenario classes (non-moral: HC, 0.28 ± 0.1; CTL, −0.26 ± 0.2; *t*_(52)_ = 3.9, *p* = 0.001, Cohen's *d* = 3.4; impersonal: HC, 0.36 ± 0.2; CTL, −0.11 ± 0.2; *t*_(52)_ = 3.4, *p* = 0.005, Cohen's *d* = 1.3; personal low: HC, 0.63 ± 0.3; CTL, 0.21 ± 0.4; *t*_(52)_ = 0.1, *p* = 0.013, Cohen's *d* = 1.2; personal high: HC, 0.68 ± 0.3; CTL, −0.01 ± 0.2; *t*_(52)_ = 4.6, *p* < 0.001, Cohen's *d* = 3.1). These finding indicate that both groups show the same overall pattern, namely increased emotional arousal to personal high-conflict scenarios. However, patients with hippocampal damage had generally increased skin conductance levels.

### Debriefing

No participant reported any difficulty in keeping track of the scenarios, and this accords with the behavioral responses being similar for both patients and controls for non-moral, impersonal, and person low-conflict scenarios.

**Figure 4.**
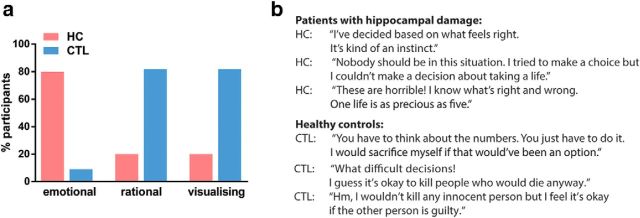
Strategies and example responses. ***a***, Percentage of patients with hippocampal damage (red) and healthy control participants (blue) who responded “yes” to debriefing questions for the personal high-conflict scenarios. The majority of patients with hippocampal damage responded to the high-conflict scenarios based on an emotional rejection of causing harm to even one person. The majority of controls used strategies that additionally took account of rational issues pertaining to future benefits when deciding in which cases it would be excusable to harm somebody. Moreover, whereas the majority of patients with hippocampal damage did not visualize the high-conflict scenarios in greater detail than any of the other scenarios, control participants visualized vivid scenes in greater detail for the high-conflict than any other scenario classes. ***b***, Examples of strategies used by the patients and healthy controls.

The responses of patients and controls did differ, however, in the strategies they used to answer the personal high-conflict scenarios (Fig. 4). Whereas controls reported an initial emotional response of how terrible it would be to kill a person, they were then able to foresee the rational benefits of saving multiple lives in the long run. In contrast, patients with hippocampal damage reported an instinctive emotional aversion to harming anybody. In addition, controls typically created an elaborate theory over the course of the task on how to decide on these conflicts, such as approving of the utilitarian option if the person to be sacrificed was a fatally wounded soldier or if you are an elected leader whose role it is to oversee the well being of others. The patients, on the other hand, did not report any such strategies. Their explanation of why they responded as they did was almost entirely based on gut feeling and a sense of what felt right and wrong for each scenario individually.

Interestingly, the patients reported that they did not tend to visualize the personal high-conflict scenarios. By contrast, the control participants commonly reported visualizing the scenes surrounding the scenarios, with the scenes associated with the high-conflict scenarios being particularly elaborate and visually intense.

### ISPC

**Figure 5.**
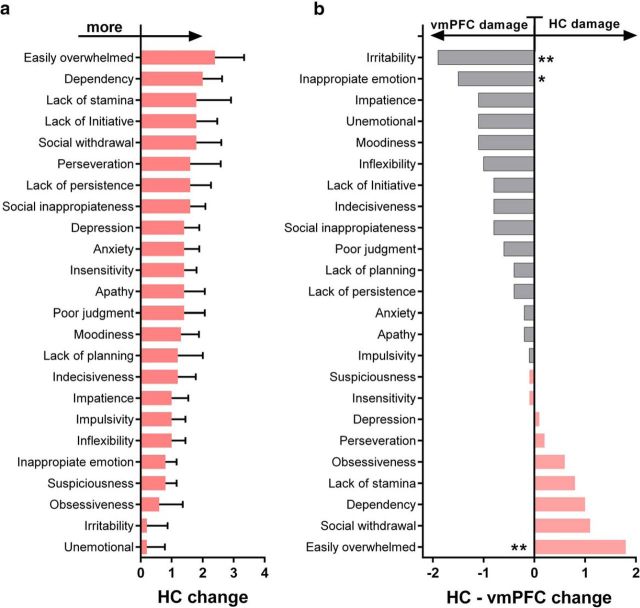
Personality changes following hippocampal damage. ***a***, Means and SEs of change scores for all 24 clinical characteristics of the ISPC for hippocampal-damaged patients. Personality characteristics are shown in decreasing order of the magnitude of change. ***b***, Difference in mean change scores for the same 24 clinical characteristics between hippocampal-damaged and previously published vmPFC-damaged (Barrash et al., 2000) patients. Characteristics are displayed in increasing order of the difference between both patient groups in this exploratory analysis. Changes greater in patients with vmPFC damage are depicted in gray; changes greater in hippocampal-damaged patients are displayed in red. *p < 0.05, **p < 0.01. Compared to patients with hippocampal lesions, patients with vmPFC damage tend to be more irritable and less emotional. By contrast, hippocampal damage leaves patients feeling more easily overwhelmed than patients with vmPFC damage.

Questionnaire responses from close relatives of all five patients were included in the analysis. All relatives knew the to-be-rated patient very well (mean years knowing the patient, 32.4 ± 10.1). Figure 5*a* illustrates the findings from this questionnaire.

Of all 28 scales that assessed a change from before to after onset of illness, the five personality characteristics that changed the most in patients with bilateral hippocampal damage were as follows: (1) easily overwhelmed (mean 2.4 ± 2.1); (2) dependency (mean 2.0 ± 1.4); (3) lack of stamina (mean 1.8 ± 2.5); (4) lack of initiative (mean 1.8 ± 1.5); and (5) social withdrawal (mean 1.8 ± 1.8).

We were also interested in exploring behavioral changes following hippocampal damage compared with vmPFC damage. We therefore examined the difference in change for all personality characteristics between our patients with hippocampal damage and already published data from patients with vmPFC damage [Barrash et al., 2000, 2011; vmPFC patients: *n* = 7; 5 males; mean age, 59.0 years (SD, 12.4); mean years of education, 11.4 (SD 2.0)]. We found a significant interaction effect between change scores for the two patient groups (*F*_(23,240)_ = 1.6, *p* = 0.046). Figure 5*b* illustrates these differences in three main parts for all 24 scales previously found to be sensitive to vmPFC damage.

The first part depicts changes that are greater following vmPFC damage than following hippocampal damage. These include irritability (HC, 0.2 ± 1.5; vmPFC, 2.1 ± 0.4; *t*_(240)_ = 2.7, *p* = 0.007, Cohen's *d* = 1.7) and inappropriate emotion (HC, 0.8 ± 0.8; vmPFC, 2.3 ± 0.8; *t*_(240)_ = 2.2, *p* = 0.033, Cohen's *d* = 1.8). The second, largest part of the graph (Fig. 5*b*) illustrates personality characteristics that did not differ between both patient groups and therefore appear to change following damage to either the hippocampus or vmPFC. This is an interesting finding, since some of the scales tap social and emotional functioning, including insensitivity, impulsivity, and lack of initiative, that have not been reported before to change following hippocampal damage. The third part of the graph illustrates personality characteristics that change in patients with hippocampal damage beyond those previously found changed in patients with vmPFC damage. These include scales indicating a tendency to become easily overwhelmed (HC, 2.4 ± 2.1; vmPFC, 0.6 ± 0.6; *t*_(240)_ = 2.6, *p* = 0.01, Cohen's *d* = 1.1) and a borderline effect for social withdrawal (HC, 1.8 ± 1.8; vmPFC, 0.7 ± 0.7; *t*_(240)_ = 1.7, *p* = 0.087, Cohen's *d* = 0.8). Of note, one has to keep in mind not only the exploratory nature of this analysis, but also that the scales of this questionnaire were constructed based on vmPFC pathology. Thus, the changes in personality in hippocampal-damaged patients may have very different causes. For example, patients with hippocampal damage who are aware of their memory impairment might lack initiative because they simply cannot remember what they were supposed to initiate, whereas there might be poor motivation underlying the lack of initiative in patients with vmPFC damage.

**Figure 6.**
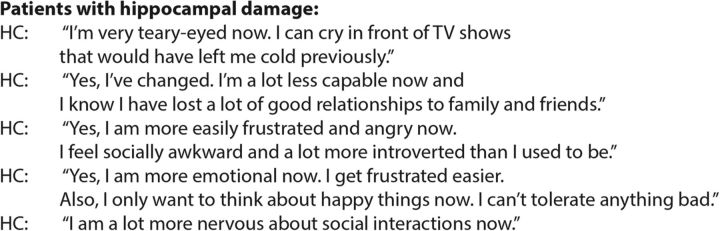
Introspective responses from patients with hippocampal damage regarding personality changes. An example response from each patient (corroborated by their relatives from the ISPC) with hippocampal damage to the question of whether they think they had changed following their illness. These answers were collected independently of the moral dilemmas task. The patients described changes in emotionality and social interactions.

Importantly for our study, patients' relatives perceived significant personality changes following hippocampal damage, especially changes related to emotional and social interpersonal interactions. This interpretation is further supported by statements made by the hippocampal-damaged patients themselves; Figure 6 includes a statement from each patient that was independently corroborated by their relatives from the ISPC. Upon asking how they think they have changed, patients most often answered that they are now more emotional and socially nervous.

## Discussion

The hippocampus and vmPFC are strongly connected both anatomically and functionally (Andrews-Hanna et al., 2010; Catani et al., 2012, 2013), and they commonly coactivate during fMRI tasks that require construction of mental scenes, such as thinking about the past or future (Addis et al., 2007), and navigation (Spreng et al., 2009). One way to try to dissociate their functional roles is to administer tasks typically associated with one structure to patients with selective damage to the other. The main goal of this study was therefore to examine how patients with selective bilateral hippocampal damage respond on a task widely associated with the vmPFC, namely moral decision making. We found that patients with hippocampal damage showed significantly reduced utilitarian responses on personal high-conflict scenarios compared with both matched healthy control participants and previously studied patients with vmPFC damage (Koenigs et al., 2007). Response rates for all other types of scenarios did not differ between the hippocampal-damaged patients and controls, indicating the patients were able to keep relevant information in mind and understood the rationale of the scenarios. This finding accords with extant literature demonstrating that patients with hippocampal damage could think rationally in situations requiring counterfactual alternatives (Mullally and Maguire, 2014) and simulating the mental states of others (Rosenbaum et al., 2007).

The dual-process theory of moral judgments posits that for a personal dilemma, two psychological systems, emotion and cognition, are pitched against each other, leading to an internal conflict situation (Greene et al., 2004). This conflict causes an initial adverse emotional response to the proposed terrible action (i.e., killing somebody), followed by a cognitive re-evaluation of the future benefits associated with that action (i.e., saving lives) and, in the end, this leads to an informed coherent emotional-cognitive decision. This is precisely what control participants described in the current study.

To map this complex process to specific brain regions, the dual-process theory suggests that the vmPFC is necessary to register or integrate the adverse emotional response into a coherent decision (Greene et al., 2004; Greene, 2007; Shenhav and Greene, 2014), leading patients with damage to that region to make more utilitarian responses. In agreement with this model, the skin conductance of vmPFC-damaged patients did not increase in response to personal scenarios (Moretto et al., 2010). Unfortunately, there are no data on the cognitive strategies used by vmPFC-damaged patients to aid in understanding why they choose the utilitarian option.

Interestingly, we found the opposite effect in hippocampal-damaged patients; that is, significantly fewer utilitarian responses and an increased emotional response to the personal high-conflict moral dilemmas. The patients showed the same pattern of skin conductance response as control participants, but overall it was increased. Since this patient population typically shows normal autonomic responses (Bechara et al., 1995; Gutbrod et al., 2006), and the amygdala volumes of our patients did not differ from those of the control participants, our finding seems indicative of a task-related emotional response and not merely a general autonomic or emotional dysfunction. Consistent with this finding, the decision-making strategies used by patients were based on an emotional, instinctive gut feeling not to harm anybody. It appears that patients got stuck in the emotional aspect of the dilemmas and were unable to overcome the initial adverse response and move on to re-evaluating and visualizing the bigger picture that would include saving multiple lives.

There is scant information about prolonged or intensified immediate emotionality in hippocampal-damaged patients. However, there is some evidence that compared with controls, negative emotions linger longer in such patients (Feinstein et al., 2010). Further, hippocampal-damaged patients designated morally bad actions as significantly worse compared with patients with vmPFC damage (Croft et al., 2010). It appears, therefore, that an immediate adverse emotional reaction to undesirable actions might become overwhelming for patients with hippocampal damage. This conclusion accords with the feedback on the ISPC from close relatives. They rated the patients as being more easily overwhelmed and more dependent than they were premorbidly. In contrast to patients with vmPFC damage, it is interesting to note that the hippocampal-damaged patients seemed to become more emotional after their illness. Notably, this was confirmed by patients themselves, with the majority mentioning increased emotionality or social nervousness when asked whether they had changed from before their illness.

We believe the increased emotionality of the hippocampal-damaged patients is reactive rather than primary. Unlike patients with vmPFC damage, patients with hippocampal damage typically do not lack insight into their illness state. All of the patients we tested knew they had a memory problem. In addition, previous research concludes that the role of the hippocampus, while possibly involved in anxiety responses, is not essential for primary emotional functioning (Clark and Maguire, 2016). However, what is clear across a number of different cognitive domains is that patients with hippocampal damage have a reduced ability to mentally construct spatially coherent scenes (Maguire and Mullally, 2013; Clark and Maguire, 2016; Zeidman and Maguire, 2016). In line with this, the hippocampal-damaged patients tested here did not visualize the high-conflict scenarios in their imagination as often as control participants. Although a decreased capacity to visualize scenes could potentially lead to over-rationalizing and hence to more utilitarian responses (Amit and Greene, 2012), in fact, visualization in healthy controls might serve to overcome the negative emotional response, enabling controls to imagine the future in which they would save multiple lives. Thus, this reduced ability to imagine scenes in the patients may have led to a dependency on a gut reaction in emotionally challenging situations that was manifested as deontological, rather than an informed global and flexibly achieved decision. This interpretation accords with recent models of morality in which deontological judgments are viewed as based on rigid decision-making processes (model-free) that depend solely on previously learned experiences (Crockett, 2013; Cushman, 2013).

Often, real-life situations and those tested in the laboratory, such as counterfactual thinking (Mullally and Maguire, 2014) or theory of mind (Rosenbaum et al., 2007), are not very emotionally challenging, and in those circumstances patients with hippocampal damage might be able to rely on both systems, emotion and cognition, equally well. For example, Craver et al. (2016) did not find a deontological bias in patients whose damage involved, but was not limited to, the hippocampi (i.e., they may have had amygdala damage). They used a moral dilemmas paradigm where participants were asked “what is morally acceptable,” potentially detracting from the emotional intensity, and hence provoking more abstract or rational responses. However, in the highly emotionally charged context of the personal moral dilemmas tested in the current study, patients with selective bilateral hippocampal damage had to answer “what would you do” in very difficult moral situations. This might have caused them to become overwhelmed with emotions because they could not imagine anything beyond immediately harming this one person. Hence, they responded in an understandable manner by stepping back and avoiding any kind of harmful action (O'Neil et al., 2015). This does not exclude the possibility that the patients' emotional profile was influenced by structural/functional connectivity changes between the hippocampus and such areas as the amygdala even in the context, as here, of normal amygdala volumes.

It is interesting to note that, like patients with hippocampal damage (Hassabis et al., 2007; Mullally et al., 2012), patients with vmPFC damage also seem to have difficulty constructing spatially coherent scenes (Bertossi et al., 2016). It could, therefore, be that patients with vmPFC damage suffer a double hit on the moral dilemmas task. That is, they lose the ability to vividly visualize the scenario and what it would mean to harm a person but also to save others, and additionally they cannot detect the adverse emotional reaction that comes with the prospect of harming a person. For them, the personal high-conflict scenarios might, in the end, be simply reduced to a numbers game of maximizing the number of survivors. By contrast, patients with hippocampal damage have a reduced ability to visualize the scenarios but the emotional reaction is preserved and presumably facilitated by their intact vmPFC. In the future, it would be interesting to examine whether hippocampal-damaged patients' moral dilemma responses “normalize” if information is provided in an already visualized form and is available throughout a task.

In summary, we found the opposite effect to the commonly reported utilitarian bias in vmPFC-damaged patients when we tested patients with bilateral hippocampal damage; that is, a strong deontological bias not to harm anybody. We suggest that visualization is used by healthy controls to make an overall informed decision on these moral dilemmas, and if this ability is impaired, one is forced to react to whatever signals remain. In the case of the hippocampal-damaged patients, this is the adverse emotional reaction to harming somebody. If this emotional reaction is also impaired, as in patients with vmPFC damage, what is left is a calculated judgment based solely on the greatest number of survivors. Overall, these results provide new insights into the processes involved in moral decision making and highlight the complementary roles played by two closely connected brain regions.
